# Vehicle re-identification method based on multi-attribute dense linking network combined with distance control module

**DOI:** 10.3389/fnbot.2023.1294211

**Published:** 2024-01-05

**Authors:** Xiaoming Sun, Yan Chen, Yan Duan, Yongliang Wang, Junkai Zhang, Bochao Su, Li Li

**Affiliations:** ^1^Heilongjiang Province Key Laboratory of Laser Spectroscopy Technology and Application, Harbin University of Science and Technology, Harbin, China; ^2^Institute of Intelligent Manufacturing Technology, Shenzhen Polytechnic, Shenzhen, China; ^3^School of Mathematics, Harbin Institute of Technology, Harbin, China

**Keywords:** vehicle re-identification, multi-attributes, HSV color space, dense connection network, distance control module

## Abstract

**Introduction:**

Vehicle re-identification is a crucial task in intelligent transportation systems, presenting enduring challenges. The primary challenge involves the inefficiency of vehicle re-identification, necessitating substantial time for recognition within extensive datasets. A secondary challenge arises from notable image variations of the same vehicle due to differing shooting angles, lighting conditions, and diverse camera equipment, leading to reduced accuracy. This paper aims to enhance vehicle re-identification performance by proficiently extracting color and category information using a multi-attribute dense connection network, complemented by a distance control module.

**Methods:**

We propose an integrated vehicle re-identification approach that combines a multi-attribute dense connection network with a distance control module. By merging a multi-attribute dense connection network that encompasses vehicle HSV color attributes and type attributes, we improve classification rates. The integration of the distance control module widens inter-class distances, diminishes intra-class distances, and boosts vehicle re-identification accuracy.

**Results:**

To validate the feasibility of our approach, we conducted experiments using multiple vehicle re-identification datasets. We measured various quantitative metrics, including accuracy, mean average precision, and rank-n. Experimental results indicate a significant enhancement in the performance of our method in vehicle re-identification tasks.

**Discussion:**

The findings of this study provide valuable insights into the application of multi-attribute neural networks and deep learning in the field of vehicle re-identification. By effectively extracting color information from the HSV color space and vehicle category information using a multi-attribute dense connection network, coupled with the utilization of a distance control module to process vehicle features, our approach demonstrates improved performance in vehicle re-identification tasks, contributing to the advancement of smart city systems.

## 1 Introduction

To strengthen road traffic management, the coverage rate of urban road traffic monitoring is increasing, resulting in the daily generation of more video image data. As the volume of video data reaches a certain threshold, the deployment of personnel for monitoring and control becomes inadequate. Consequently, vehicle recognition technology has been introduced. Vehicle re-identification aims to identify the same vehicle in different locations and at different times based on the vehicle information collected by a fixed-position sensor.

As early as 1998, Coifman B recalculated features, such as the effective vehicle length between two continuous metric stations on the highway (Coifman, 1998). This method is too limited; it represents an early form of vehicle re-identification. Abdulhai and Tabib (2018) attempted to improve the accuracy of vehicle re-identification at continuous loop detection stations by enhancing the mode proximity distance metric in the pattern recognition process. Relevant experiments were not completed but showed the potential to enhance the accuracy. Liu et al. (2016a) created a vehicle re-identification (VeRi) dataset based on real urban surveillance scenes. Since then, research in re-identification has progressed rapidly. Zhu et al. (2018) proposed a joint deep learning method (JFSDL) for vehicle re-identification. The Siamese Deep Network is used to extract the features of the input vehicle image pairs, and the similarity score between the input vehicle image pairs is obtained based on the hybrid similarity learning function. Lou et al. (2019a) created a new super large vehicle re-identification dataset VERI-wild, which contains more than 400,000 images of 40,000 vehicles. Zheng et al. (2021) used four public vehicle datasets to create a unique large vehicle dataset called VehicleNet and developed a two-step progressive approach to learn more robust visual representations from VehicleNet. Qian et al. (2020) proposed a deep convolutional neural network (SAN) based on dual branching and attribute perception to learn effective feature embedding for vehicle recognition tasks. Ratnesh et al. (2020) used triplet embedding to solve the problem of vehicle re-identification *in camera* networks. Peng et al. (2020) proposed an adaptive vehicle re-identification domain adaptive framework (DAVR) that uses the tag data from the source domain to adapt to the target domain, reducing cross-domain bias. Teng et al. (2020) proposed a multiview branch network, where each branch learns a view-specific feature and introduces a spatial attention model into each feature-learning branch to strengthen the ability to discriminate local differences. Jin et al. (2021) proposed a multicentric metric learning method for vehicle re-identification in multiple views. Zhang et al. (2022) proposed a double attention granularity network (DAG-Net) for vehicle re-identification. The dual-branch neural network was used to extract coarse-grained and fine-grained features, and a self-attention model was added to each branch to enable DAG-Net to recognize different regions of interest (ROIs) at coarse and fine levels for coarse-grained and fine-grained identification. Subsequently, Guo et al. (2019) proposed a novel two-stage attention network supervised by the Top-k Accuracy Multiple Granularity Ranking Loss (TAMR), aiming to learn effective feature embedding for the vehicle re-identification task. Hou et al. (2019) introduced the Deep Quadruplet-wise Adaptive Learning method (DQAL), which introduces the concept of quadruplets and generates four sets of inputs. By combining the proposed quadruplet network loss and softmax loss, they developed a quadruplet network to learn more discriminative vehicle recognition features. Zhang et al. (2019) introduced the Partial Guidance Attention Network (PGAN), effectively integrating global and partial information for discriminative feature learning. Bashir et al. (2019) took the pioneering approach of addressing vehicle re-identification in an unsupervised manner, utilizing a progressive two-step cascaded framework to formulate the entire vehicle re-identification problem as an unsupervised learning paradigm. PAMAL (Tumrani et al., 2020) utilized multi-attribute features, i.e., color and type, and vehicle key points to solve the re-identification task. MSCL (Yuefeng et al., 2022) achieves unsupervised vehicle re-identification through the integration of the Discrete Sample Separation module and Mixed Sample Contrastive Learning. VAAG (Tumrani et al., 2023) addresses the re-identification task by learning robust discriminative features encompassing camera views, vehicle types, and vehicle colors.

In summary, an algorithm for classifying the color features of vehicles based on the HSV color space is proposed. The image is transformed into the HSV color space, and saturation (S) and brightness (V) are introduced, which are sensitive to the reflection coefficient of the object surface. The color features in the HSV color space are extracted by a feature extraction network for accurate color attribute classification. Second, based on the concepts of the YOLO model and DenseNet network, an improved densely connected vehicle classification network is designed by integrating the extracted color features in the HSV color space. The improved network model is used to obtain different dimensional features for the image of the target vehicle, reducing the amount of computation and improving the feature usage rate. The results of the different dimensional features are weighted and fused to improve the accuracy of vehicle classification. It is combined with the vehicle re-identification network to quickly propose class-independent images for the re-identification network. Based on the traditional vehicle recognition network, a new distance control block (DC module) is developed in this study. According to the feature extraction network, the features extracted from the image are processed by similarity DC or difference DC to shorten the feature distance within the image class and increase the feature distance between image classes. Finally, the performance of this algorithm is verified by experiments.

## 2 Methods

### 2.1 Multi-attribute dense link classification

**Figure 1:**
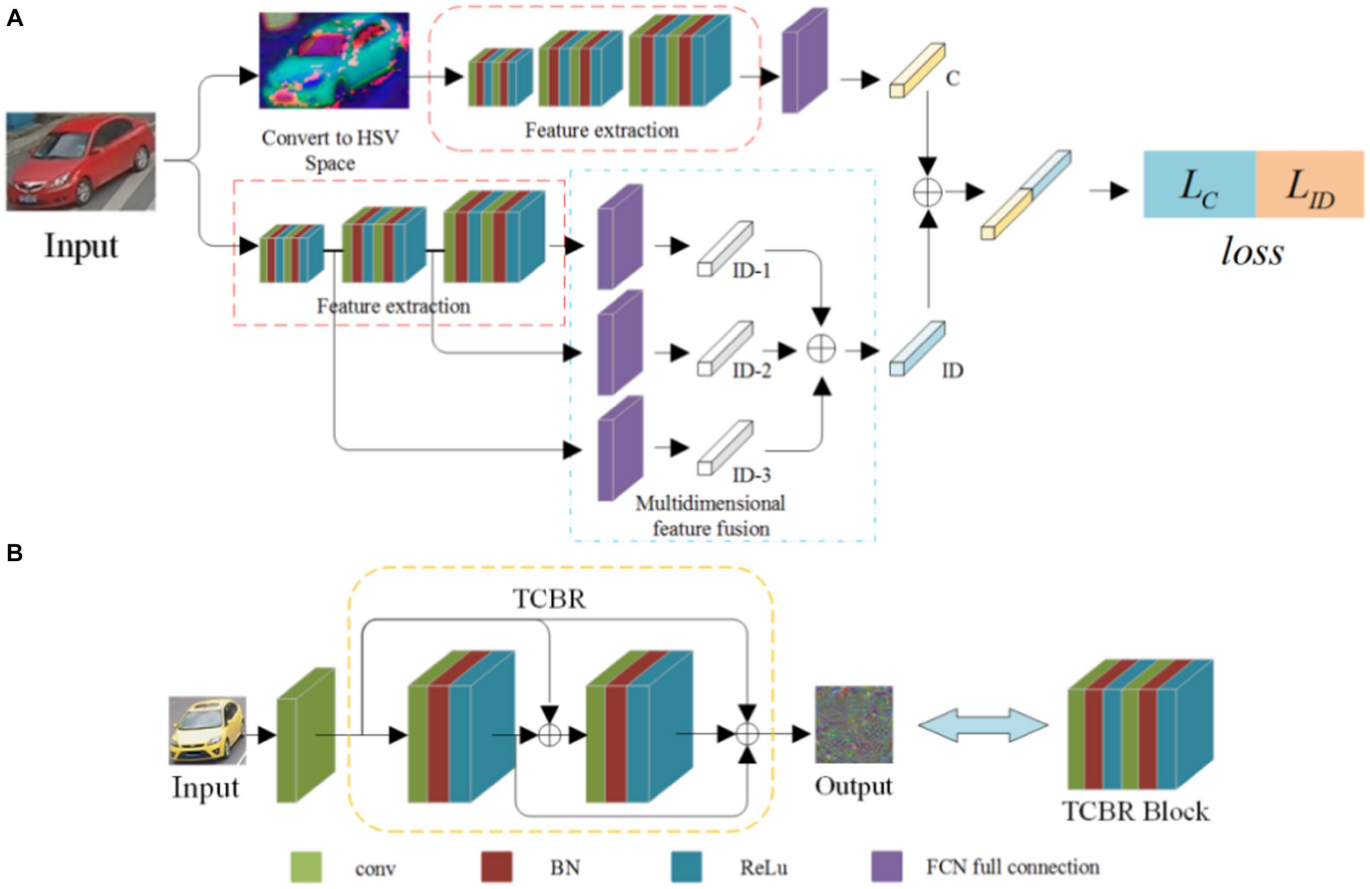
Flowchart of the classification with dense connection and multiple attributes. **(A)** Schematic diagram of the multi-attribute dense connection network structure, **(B)** Structural diagram of the small dense connecting block.

In this section, a vehicle classification method based on a dense network with multiple attributes is proposed. The test images are filtered, and the images that are similar to the target vehicle are re-recognized to eliminate the images that do not match the target vehicle class. There are many vehicle attributes, such as model, color, detail features, and volume. In this section, the classification of the dense connection of several attributes is continued, and the most characteristic model and color are selected as the research objects. This method uses a dense connectivity structure to reduce the computational overhead in the network. It combines the color features in the HSV space to minimize the impact of the external environment on vehicle color recognition. The flowchart is shown in Figure 1A. The individual steps are as follows.

#### 2.1.1 Color feature extraction

There are various colors of vehicles. In this study, the colors of vehicles are classified into 10 categories: yellow, orange, green, gray, red, blue, white, gold, brown, and black.

1. Convert the RGB image to an HSV image, as shown in Formula (1).

(1) $$\begin{array}{c} \max\left(i,j\right)=\max\left[I_{R}\left(i,j\right),I_{G}\left(i,j\right),I_{B}\left(i,j\right)\right]\\ \min\left(i,j\right)=\min\left[I_{R}\left(i,j\right),I_{G}\left(i,j\right),I_{B}\left(i,j\right)\right]\\ \Delta =\max\left(i,j\right)-\min\left(i,j\right) \end{array}$$

Where $I_{R}\left(i,j\right)$ , $I_{G}\left(i,j\right)$ , and $I_{B}\left(i,j\right)$ represent the values of the R component, G component, and B component corresponding to the pixel coordinate $\left(i,j\right)$ points, $\max\left(i,j\right)$ represents the maximum value among the R, G, and B components, $\min\left(i,j\right)$ represents the minimum value among the R, G, and B components, and Δ takes the difference between the two, representing the span of the three components.

The values of the *H* component, *S* component, and *V* component are calculated according to Formula 2. The calculation involves determining the values of the *H* component, *S* component, and *V* component in the HSV color space.


(2) $$\begin{array}{c} H\left(i,j\right)=\left\{\begin{array}{l} \left[I_{G}\left(i,j\right)-I_{B}\left(i,j\right)\right]/\Delta \times 60,\;\max\left(i,j\right)=I_{R}\left(i,j\right)\\ 120+\left[I_{B}\left(i,j\right)-I_{R}\left(i,j\right)\right]/\Delta \times 60,\max\left(i,j\right)=I_{G}\left(i,j\right)\\ 240+\left[I_{R}\left(i,j\right)-I_{G}\left(i,j\right)\right]/\Delta \times 60,\max\left(i,j\right)=I_{B}\left(i,j\right) \end{array}\right.\\ V\left(i,j\right)=\max\left(i,j\right)\\ S\left(i,j\right)=\Delta /\max\left(i,j\right) \end{array}$$

Where $H\left(i,j\right)$ , $S\left(i,j\right)$ , and $V\left(i,j\right)$ represent the values of *H* component, *S* component, and *V* component corresponding to the pixel with coordinate $\left(i,j\right)$ converted to HSV color space;

1. Color feature extraction

The structure of the feature extraction network consists of three TCBR blocks. As shown in Figure 1B, each TCBR block is composed of two CBR blocks, and each CBR block is made up of a convolution layer, a BN layer, and a ReLU layer. The TCBR block is a Twice Convolution Batch Normalization ReLU block structure. In the TCBR block, all outputs are summed before the input of the second CBR block, and the features of the input, the first output, and the double output are summed as the input of the next layer, i.e., the dense connections. The outputs of each output node are directly summed, ensuring consistent dimensions for the results of each output node. This reduces the computation of the network, and the dimension conversion is achieved by adding a convolutional layer between two TCBR blocks, making the network more flexible.

The feature extraction network is shown in Figure 1A. Each TCBR block performs a dimensional transformation through the convolutional layer and the pooling layer, extracting features of different dimensions to obtain high-dimensional features of the image.

The extracted high-dimensional features are fed into the fully connected layer, mapping the features to the sample space. Subsequently, the color feature vector *C* corresponding to the HSV features is obtained through regression.

#### 2.1.2 Extraction of the category characteristics

**Figure 2:**
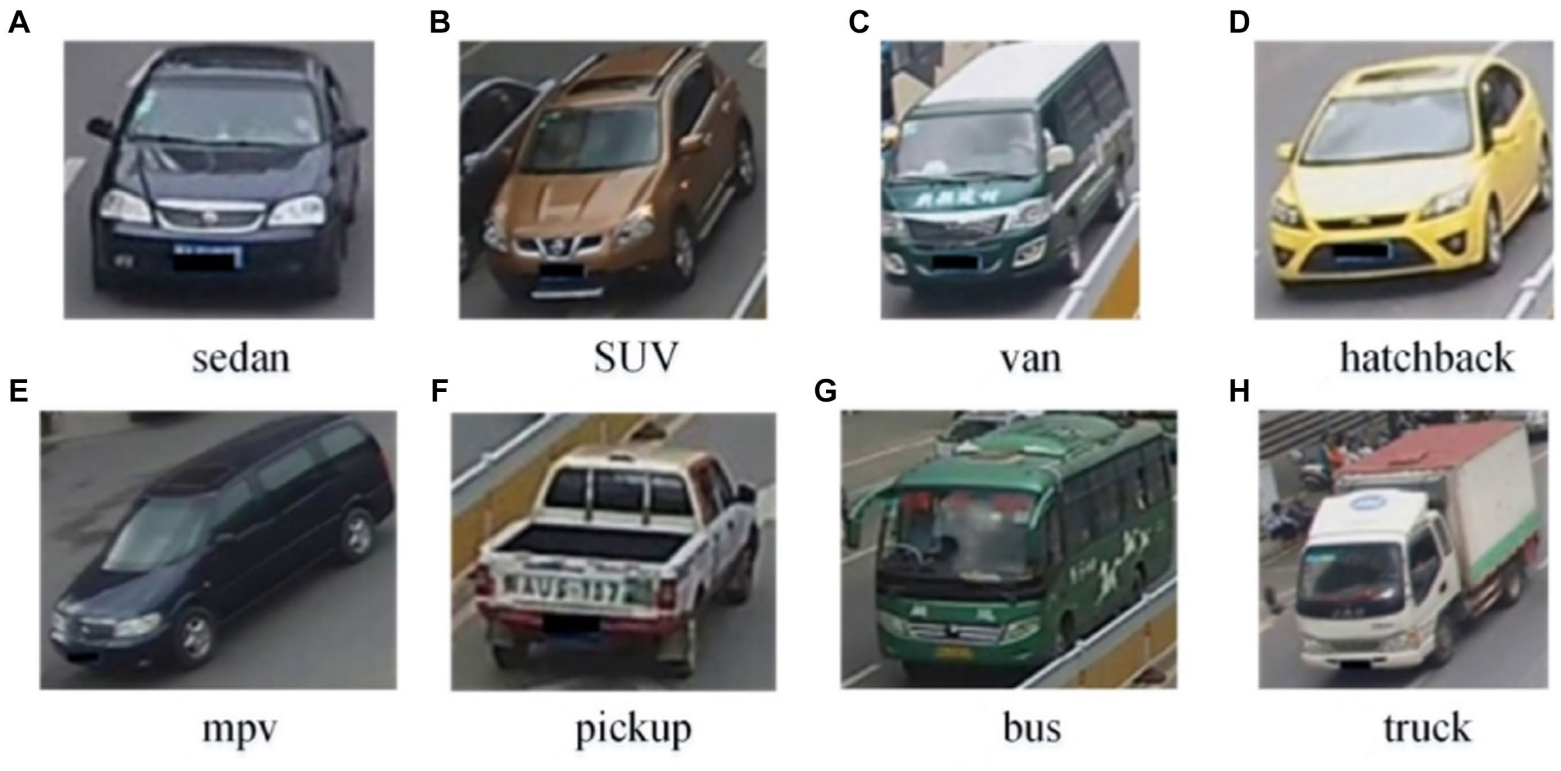
Example images of eight vehicle types. **(A)** sedan; **(B)** SUV; **(C)** van; **(D)** hatchback; **(E)** mpv; **(F)** pickup; **(G)** bus; **(H)** truck.

In this study, vehicles are classified into eight categories. The category designations from 1 to 8 are sedan, SUV, van, hatchback, MPV, pickup, bus, and truck. Figure 2 shows schematic representations of these eight vehicle types.

1. Multidimensional Feature Extraction

In multi-dimensional feature extraction, the TCBR module mentioned above is utilized to extract multi-dimensional features. To better eliminate various features of the vehicle, the feature extraction network is correspondingly improved, as shown in Figure 1. Three different dimensions of features are extracted, namely, ID−1 , ID−2 , and ID−3 .

1. Multi-dimension feature fusion

According to Formula 3, the three eigenvectors ( ID−1 , ID−2 , and ID−3 ) are:


(3) $$\begin{array}{l} f_{1}=\left(p_{A},p_{B},p_{C},\ldots ,p_{H}\right)\\ f_{2}=\left(p_{B},p_{A},p_{C},\ldots ,p_{H}\right)\\ f_{3}=\left(p_{C},p_{A},p_{B},\ldots ,p_{H}\right) \end{array}$$

where $p_{A}$ ( A−H corresponds to eight vehicle types) is the maximum value in $f_{1}$ , indicating that the probability of the picture being type A is the highest. Similarly, $p_{B}$ is the maximum value in $f_{2}$ , signifying that the image has the highest probability of being type *B*; $p_{C}$ is the maximum value in $f_{3}$ , indicating that the image has the highest probability of being class *C*. The classes A, B, and C are distinguished for better comprehension. In practice, these classes (A, B, and C) can be the same.

Then, the scores are calculated. For type A, as shown in Formula (4).


(4) $$w_{A1}=\frac{f_{1}\left(p_{A}\right)}{f_{1}\left(p_{A}\right)+f_{2}\left(p_{A}\right)+f_{3}\left(p_{A}\right)}$$

Where $w_{A1}$ is the weight coefficient of $p_{A}$ in the weight value of type *A* in the vector $f_{1}$ . Similarly, $w_{A2}$ and $w_{A3}$ can be obtained. As shown in Formula (5):


(5) $$\begin{array}{l} w_{A2}=\frac{f_{2}\left(p_{A}\right)}{f_{1}\left(p_{A}\right)+f_{2}\left(p_{A}\right)+f_{3}\left(p_{A}\right)}\\ w_{A3}=\frac{f_{3}\left(p_{A}\right)}{f_{1}\left(p_{A}\right)+f_{2}\left(p_{A}\right)+f_{3}\left(p_{A}\right)} \end{array}$$

Finally, the score $S_{A}$ of type *A* is as shown in Formula (6).


(6) $$S_{A}=\left[{w_{A1}}^{*}f_{1}\left(p_{A}\right)+{w_{A2}}^{*}f_{2}\left(p_{A}\right)+{w_{A3}}^{*}f_{3}\left(p_{A}\right)\right]$$

As a result, the values $S_{B}$ and $S_{C}$ of type *B* and type *C* are determined in the same way. One compares three values and takes the highest corresponding type as the classification result.

#### 2.1.3 Multi-attribute dense connection classification

The *output* of the feature classification network is a one-dimensional vector, as shown in Formula (7).


(7) $$\mathrm{output}=\left[C,\mathrm{ID}\right]$$

where *C* represents the color feature information in the HSV space of the vehicle in the image, which is a $\mathrm{one}-\mathrm{hot}\left(10\right)$ vector, representing the normalized value of the ratings corresponding to the 10 color categories; *ID* represents the class feature information of the vehicle in the image, which is a $\mathrm{one}-\mathrm{hot}\left(8\right)$ vector, representing the normalized value of the ratings corresponding to the eight vehicle categories.

#### 2.1.4 Loss function

The network receives the color feature information and the vehicle category feature information simultaneously, so the loss function also has two parts, namely, the color feature loss and the vehicle category feature loss. The loss function is developed based on the cross-entropy loss. The loss function for color features is shown in Formula (8).


(8) $$L_{C}=-\overset{n}{\underset{i=1}{\sum }}q\left(i\right)\log\left(p\left(i\right)\right)$$

where *n* represents the number of color categories and assigns the color attribute to the color attribute category. $p\left(i\right)$ represents the probability that the image belongs to category *i*, $q\left(i\right)$ is a symbolic function. If category *i* is a basic category, the value is 1, and if category *i* is not a real category, the value is 0.

The loss function $L_{\mathrm{ID}}$ of vehicle category characteristic loss is as shown in Formula (9).


(9) $$L_{\mathrm{ID}}=-\overset{m}{\underset{j=1}{\sum }}q\left(j\right)\log\left(p\left(j\right)\right)$$

where *m* is the number of categories of vehicle, which was given before. There are eight types, $p\left(j\right)$ denotes the probability that the image belongs to type *j*, and $q\left(j\right)$ is also a symbolic function. If the category *j* is an objective type, the value is 1, and if the category *j* is not an objective type, the value is 0.

The final network *loss* function *loss* is shown in Formula (10):


(10) $$\mathrm{loss}=L_{C}+L_{\mathrm{ID}}$$

### 2.2 Vehicle re-identification

**Figure 3:**
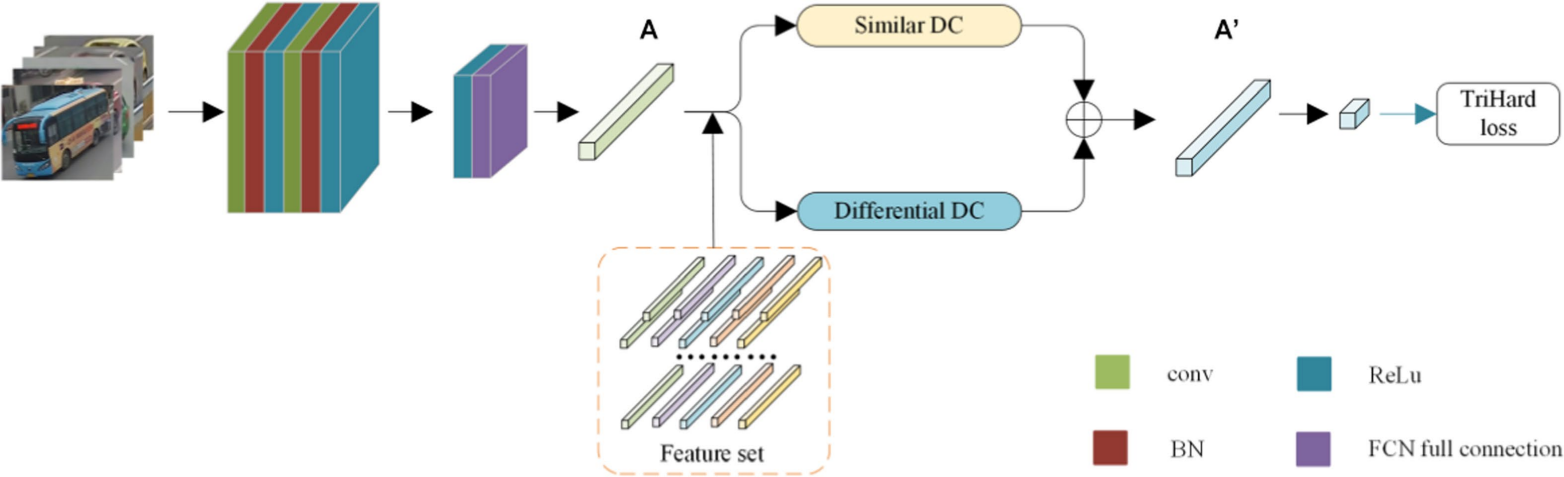
Vehicle re-identification model based on DC module.

After classifying the dense connection of multiple attributes, the system algorithm has filtered out the vehicles with the same color and category as the query vehicle. Then, the final process of re-identifying the vehicle is performed. As shown in Figure 3, the network first extracts features from the input image and obtains high-dimensional features *A*. Then, based on the features of the same category in the feature set, the feature *A* is processed by a similarity DC module and a differential DC module, and the two features are merged into a new feature $A^{\prime }$ .

#### 2.2.1 Feature extraction

The function of the feature extraction network is to extract the high-dimensional features of the input images. To reduce the computational cost, the TCBR module proposed in Chapter 3 is used. As shown in Figure 3, it can be observed that the network consists of two cascaded TCBR modules, and the convolutional layer in the middle is used for dimension conversion. The shape of the input image is set to 224 × 224. First, the convolution layer with the size of the fusion kernel b, the number of fusion kernels 50, and the step size 1 is introduced to perform the pre-convolution. Then, the first TCBR module is instructed to perform dense convolution with multiple inputs in 50 dimensions. Moreover, a convolution layer with the size of the convolution kernel 3 × 3, the number of convolution kernels 100, and the step size 1 is introduced to perform the dimension transformation. As the last step, the second TCBR module is instructed to perform the convolution of features with multiple inputs in 100 dimensions, and then the feature map is output.

#### 2.2.2 Feature set production

The feature set corresponds to the training set used, and one image is selected from each category. Assuming that the total number of classes is the same, the feature extraction network from the previous section is used to extract the features, and the extracted features are integrated into the feature set. The feature set is the feature vector cluster with the category number.

#### 2.2.3 DC module processing

Introduction of DC module: the DC module is divided into two types, one is the similarity module DC, which is based on the target image and uses the comparison image for similarity pooling; the other is the difference module DC, which is based on the target image and performs difference pooling of the contrast image. Each point in the high-dimensional feature space represents the corresponding semantic features of that part. That is, in a sense, they are the domain features. For the similarity module DC, the image after processing attenuates the influence of the prominent features (e.g., the lamp and window position features are identical to the target image).

In contrast, after processing in the DC module, the image may enhance the influence of secondary features (such as body and other parts). For the positive sample (i.e., the image belonging to the same vehicle as the target image), the influence of secondary features is greater than in the negative sample. After two DC modules, the distance between the target image and the positive example is “close.” For negative examples, the secondary feature itself is smaller than in positive examples. After processing two DC modules, the influence of secondary features, “pulling away” and distance of the target image, is increased.

**Figure 4:**
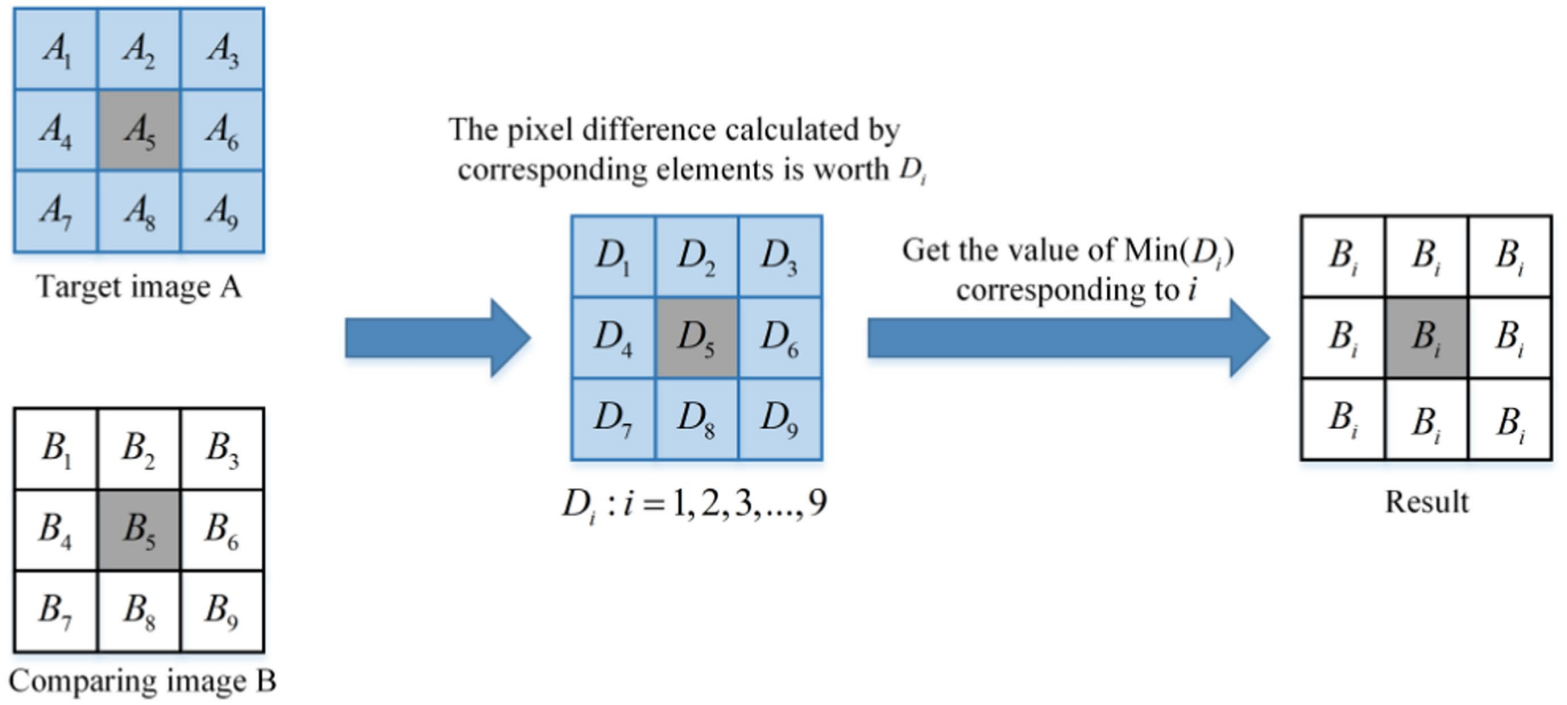
The principle of the similarity DC module.

1. Similarity DC module schematic diagram is as follows:

As shown in Figure 4, the schematic diagram of a similar DC module is presented. It can be observed from Figure 4 that the core size of the module is 3 × 3. After extracting the input sample pair, the feature map is traversed by a window of size 3 × 3, with a step size of 2. The difference value of the corresponding pixels in the window is calculated, and the pixel value corresponding to the minimum difference value is selected to replace the pixel value of the points in the window. The image is divided into a small window of size 3 × 3. $A_{i}\left(i=1,2,3,\ldots ,9\right)$ is used to represent the values of 9 points in the target image *A* window, and $B_{i}\left(i=1,2,3,\ldots ,9\right)$ is used to describe the values of 9 points in the contrasting image *B* window. The distance $D_{i}\left(i=1,2,3,\ldots ,9\right)$ between the corresponding points is calculated, as shown in Formula (11).


(11) $$D_{i}=\left|A_{i}-B_{i}\right|\;\left(i=1,2,3,\ldots ,9\right)$$

The minimum value $D_{\text{min}}$ in $D_{i}$ is obtained as shown in Formula (12).


(12) $$D_{\text{min}}=\min\left\{D_{1},D_{2},D_{3},\ldots ,D_{9}\right\}$$

The corresponding pixel index value *m* for $D_{\text{min}}$ is shown in Formula (13).


(13) $$m=\sum f\left(i\right)\;\left(i=1,2,3,\ldots ,9\right)$$

The *m* value obtained by Formula (13) is the index value of the nearest point in the corresponding window between the target image *A* and the contrast image *B*, and the $f\left(i\right)$ definitions are as shown in Formula (14).


(14) $$f\left(i\right)=\{\begin{array}{l} i,\;D_{i}=D_{\text{min}}\\ 0,\;D_{i}\neq D_{\text{min}} \end{array}$$

Then, the value of all points is replaced in the contrast image *B* window with the value $B_{m}$ corresponding to point *m*, as shown in Formula (15).


(15) $$B_{i}=B_{m},\;\left(i=1,2,3,\ldots ,9\right)$$

**Figure 5:**
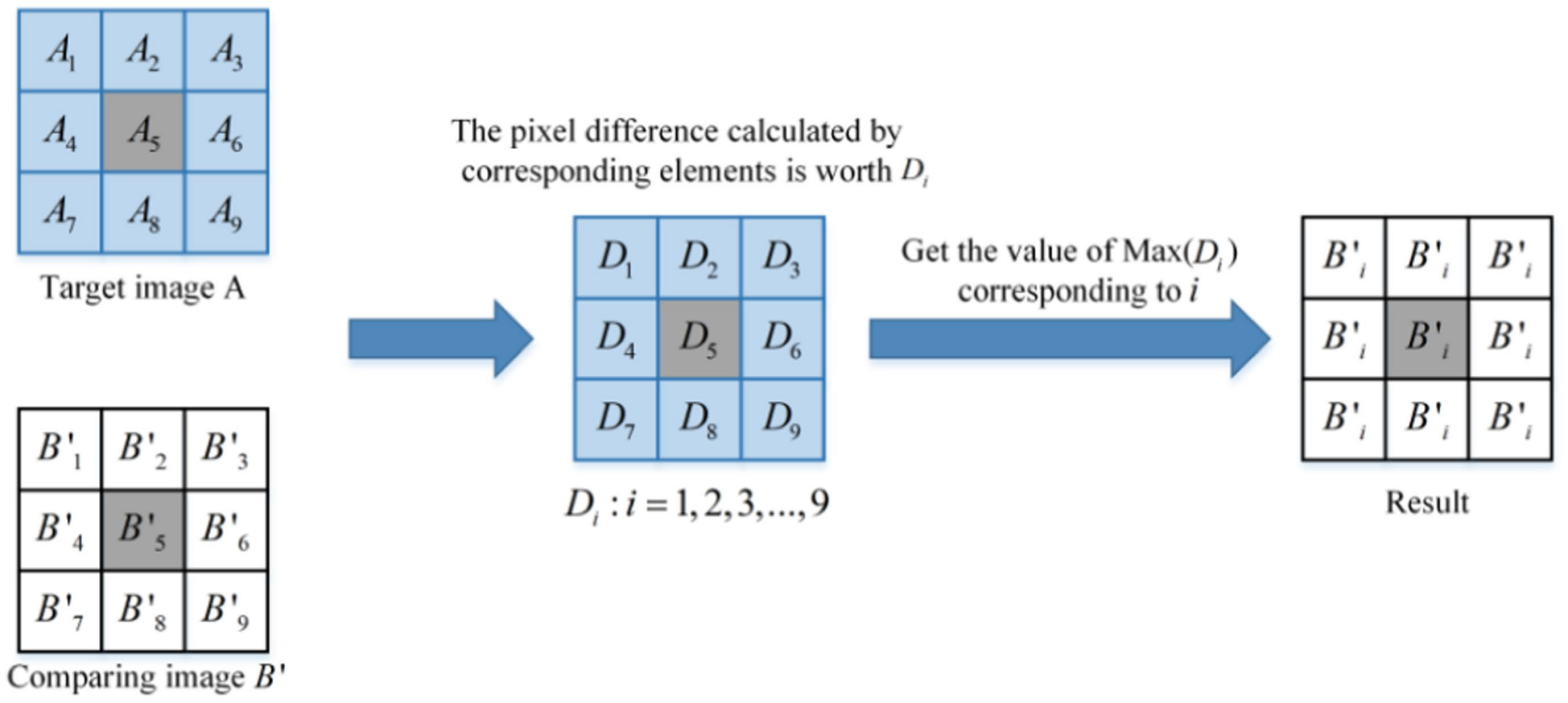
The principle of the difference DC module.

1. The schematic diagram of the different DC modules is as follows:

As shown in Figure 5, this is the schematic representation of the various DC modules. Similarly, it traverses the feature map with a window size of 3 × 3, and the step length is 2. The differences between the corresponding pixels in the window are calculated. The pixel value corresponding to the point with the greatest difference is replaced by the pixel value of the entire window.

The preceding part is similar to the aforementioned DC module. The image is divided into a small window of 3 × 3. $A_{i}\left(i=1,2,3,\ldots ,9\right)$ represents the values of nine points in the *A* window of the target image, and $B_{i}^{'}\left(i=1,2,3,\ldots ,9\right)$ represents the values of nine points in the $B^{\prime }$ window of the contrast image. The distances $D_{i}^{'}\left(i=1,2,3,\ldots ,9\right)$ between the corresponding points are then calculated, as shown in Formula (16).


(16) $$D_{i}^{'}=\left|A_{i}-B_{i}^{'}\right|\;\left(i=1,2,3,\ldots ,9\right)$$

The maximum value $D_{\text{max}}^{'}$ in $D_{i}^{'}$ is obtained as shown in Formula (17).


(17) $$D_{\text{max}}^{'}=\max\left\{D_{1}^{'},D_{2}^{'},D_{3}^{'},\ldots ,D_{9}^{'}\right\}$$

The corresponding pixel index value $m^{\prime }$ for $D_{\text{max}}^{'}$ is shown in Formula (18).


(18) $$m^{\prime }=\sum f\left(i\right)\;\left(i=1,2,3,\ldots ,9\right)$$

The value of $m^{\prime }$ obtained in Formula (18) represents the index corresponding to the farthest point within the window between the target image *A* and the contrast image $B^{\prime }$ . Here, $f\left(i\right)$ is defined as shown in Formula (19).


(19) $$f\left(i\right)=\{\begin{array}{l} i,\;D_{i}^{'}=D_{\text{max}}^{'}\\ 0,\;D_{i}^{'}\neq D_{\text{max}}^{'} \end{array}$$

Then, the values of all points in the window of contrast image $B^{\prime }$ is replaced with the value $B_{m'}$ corresponding to the point $m^{\prime }$ , as shown in Formula (20).


(20) $$B_{i}^{'}=B_{m'},\;\left(i=1,2,3,\ldots ,9\right)$$

After the sample pairs are processed, the corresponding similarity values are calculated, and the average value of the two is output as the final similarity coefficient.

For the similarity DC module, all eigenvalues in the window are replaced by the eigenvalues with the smallest distance between features in the feature map *A*. Similarly, for the differential module DC, all eigenvalues in the window are replaced by the eigenvalues with the widest distance between features in the feature map *A*. The average value of the corresponding elements in the two obtained features is then calculated to obtain the final feature $A^{\prime }$ .

#### 2.2.4 Loss function

We train the model using a training set divided into batches. Each batch contains images *P × K*, where *P* is the number of categories, and each category contains *K* images. First, three images are selected from the batches to feed the model, and *a* represents the current data, *P* is the image of the same category as *a*, and *n* is the image of a different type. Assuming that the sample is *x* and the total number of examples in the training set is *N*, the loss function *TriHard loss* is formulated in Formula (21).


(21) $$\begin{array}{c} \mathrm{TriHard}\;\mathrm{loss}=\frac{1}{N}\left\{\overset{N}{\underset{i}{\sum }}{\left[d{\left(a,p\right)}_{\text{max}}-d{\left(a,n\right)}_{\text{min}}+\alpha \right]}_{+}\right\}\\ d\left(a,p\right)=f\left(x_{i}^{a}\right)-f{\left(x_{i}^{p}\right)}_{2}^{2}\\ d\left(a,n\right)=f\left(x_{i}^{a}\right)-f{\left(x_{i}^{p}\right)}_{2}^{2} \end{array}$$

where $f\left(x\right)$ represents the mapping function of the model; max represents the maximum value; ‘min’ represents the minimum value; α represents the distance interval; the loss function makes the difference between $d\left(a,p\right)$ and $d\left(a,n\right)$ better than α .

## 3 Similarity metric

**Figure 6:**
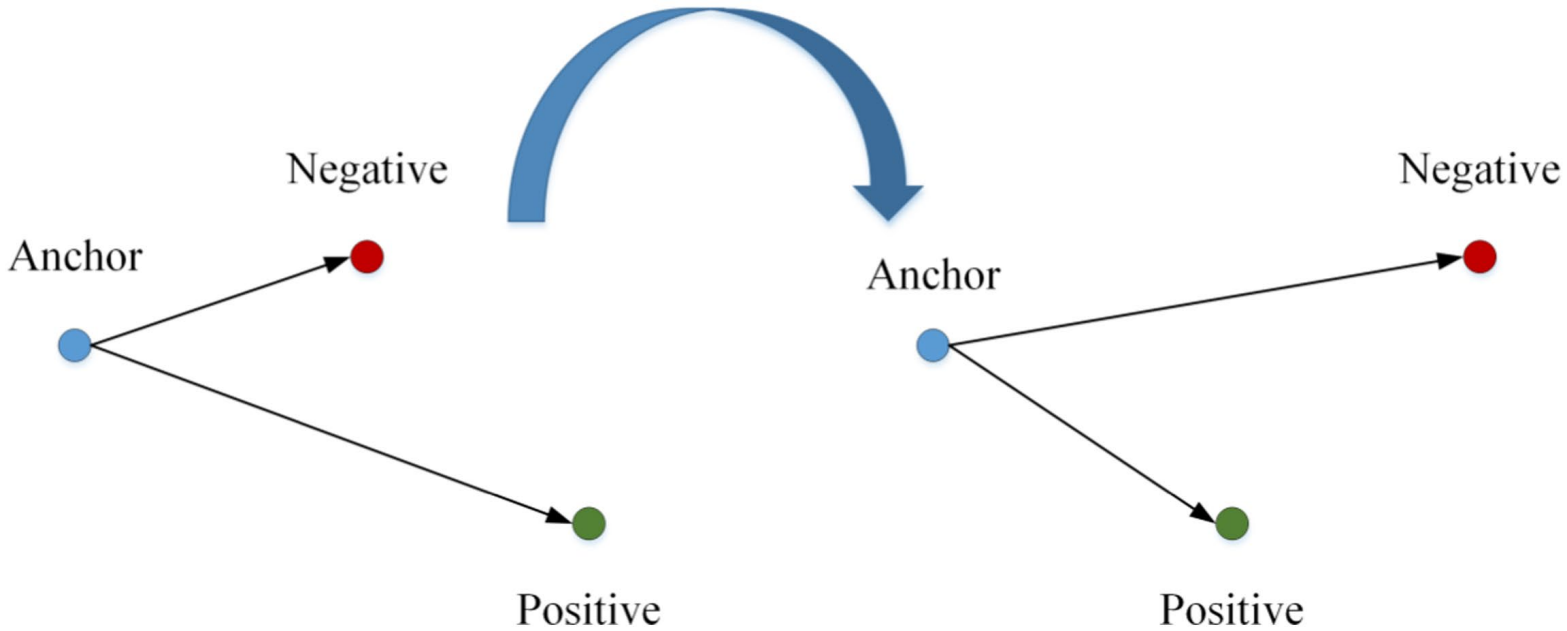
Metric learning method of triad loss function.

**Figure 7:**
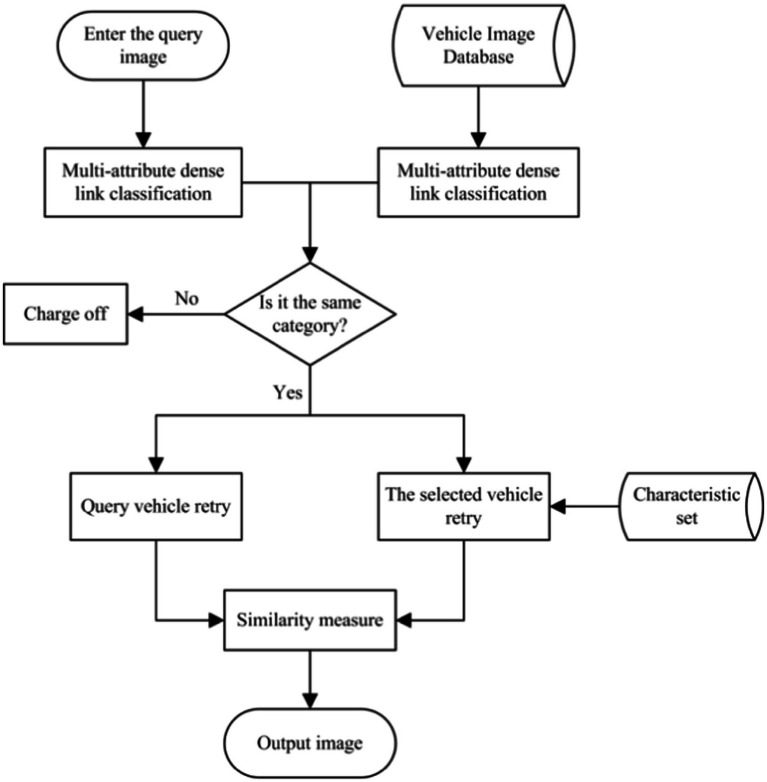
The overall flow chart of this algorithm.

The core of vehicle re-identification tasks is to find and sort vehicle images. The ideal vehicle re-identification network model can make the distance metric between images of the exact vehicle smaller and more significant. In this study, the vehicle re-identification distance metric model is trained by vehicle models. As shown in Figure 6, in this study, according to the principle of metric learning based on the triple loss function, the distance metric between the anchor and the positive sample point becomes smaller and that between the anchor and the negative sample point becomes larger by training. This way, the recognition performance of re-identification of a vehicle with a positive sample is realized. The overall flow chart of this algorithm is shown in Figure 7.

## 4 Experimental results and analysis

### 4.1 Image dataset

The image data in this article comes from the VeRi776 datasets (Liu et al., 2016a) and VeRi-Wild datasets (Lou et al., 2019b). The VeRi776 dataset contains 50,000 images of 776 vehicles captured by 20 cameras without restrictions on traffic. Images of each vehicle are captured by 2 to 18 cameras with different viewing angles, illuminations, occlusions, and resolutions. Each vehicle image is tagged with vehicle ID and vehicle type information, with vehicle category information divided into nine categories. The dataset includes both a training set and a test set. The training set contains 37,782 images of 576 vehicles; the test set contains 13,257 images of 200 vehicles. To evaluate the results, the test dataset is further divided into a vehicle image library and test vehicle images. The vehicle image library contains 11,579 images of 200 vehicles, and the test vehicle image contains 1,678 images of 200 vehicles.

**Figure 8:**
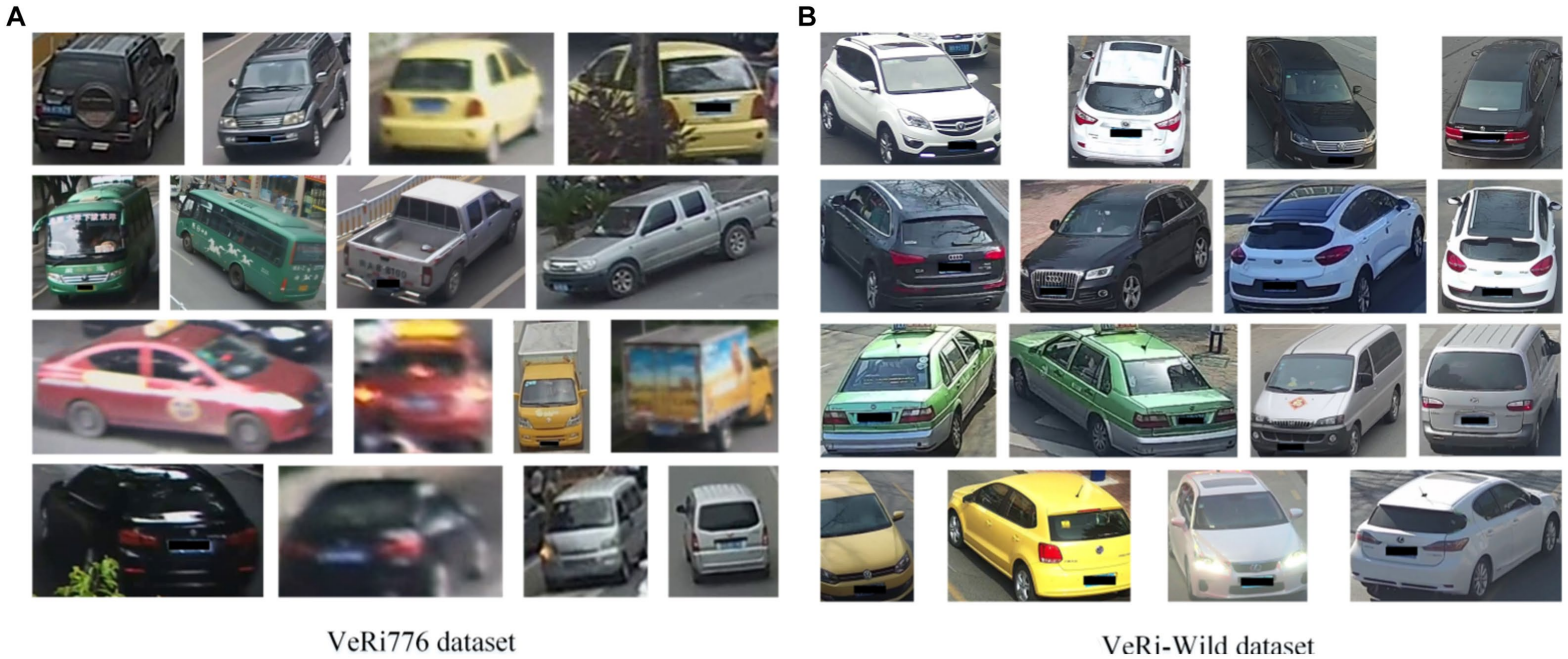
Some example images from both VeRi776 and VeRi-Wild datasets. **(A)** Example images from the VeRi776 dataset, **(B)** Example images from the VeRi-Wild dataset.

The imaging background and environmental variations of the VeRi-Wild dataset are more complex, and more camera models are used. The vehicle images in the dataset are captured by a 174-camera surveillance system covering more than 200 square kilometers. The total acquisition cost is 1 month. In total, more than 400,000 images of 40,000 vehicles were acquired (an average of 10 images per vehicle). In addition, the image angles included for the same vehicle vary widely. The dataset only annotates the vehicle ID without other information. This is the first dataset for vehicle re-identification without constraints. The sample images from the VeRi776 and VeRi-Wild datasets are shown in Figure 8.

### 4.2 Implementation details

We utilized the PyTorch framework to develop the network. The platform for training and testing is the Ubuntu 18.04 system, with a GTX 1070 Ti graphics card and 10GB of video memory. The hardware configuration for the experiments is presented in Table 1. This article uses the Adam optimizer with a learning rate of 0.001 and a momentum of 0.9. Since the neural network is very unstable at the beginning of training, a corresponding training strategy, cosine annealing learning, is added to reduce the risk of overfitting so that the model has strong robustness and good convergence to occlusion. In the cosine annealing strategy, the learning rate is reduced in the form of a cosine function, which ensures a smoother learning rate reduction and prevents the model from failing to converge because the learning rate is dropping too fast. The minimum learning rate is 0.00001.

**Table 1:** Hardware equipment of practical environment.

Laboratory equipment	Experimental configuration
System	Ubantu18.04
Deep learning framework	Pytorch
Programming language	Python
Compiler	PyCharm
Running memory	32G
CPU	$\mathrm{Inte}r^{R}Core^{TM}i7-8750H,2.20\mathrm{GHz}$
GPU	GTX1070Ti

The batch size is set to 32. We trained the network for 100 epochs. The experimental computer hardware configuration is shown in Table 1.

### 4.3 Experimental metric standard

In this experiment, rank−n , CMC curves, and mAP were used as experimental indexes to measure the model effect.

1. *Accuracy*:

*Accuracy* is shown in Formula (22).


(22) $$\mathrm{Accuracy}=\frac{p}{N}$$

where *p* is the number of correctly identified samples, and *N* is the total number of samples.

1. Classification rate *v*:

Using classification rate *v* to measure the speed of classification, the formula is shown in Formula (23).


(23) v=n/s

where *n* is the number of classified samples, and *s* is the time needed to classify these pieces.

1.
rank−n and CMC curve:

The result of vehicle re-identification is output as *n* images with the highest similarity between the test set and the query image. rank−n represents the probability that the output of the first image,

after model determination, contains the correct image. For example, rank−1 represents the probability that the image with the highest similarity output, after model determination, is the correct image. rank−5 represents the probability that the first five image outputs, after model determination, contain the correct image.

The CMC curve takes *n* value of rank−n as abscissa, and the corresponding probability that the correct images are included, which is denoted by 8.

1.
mAP (Average precision rate):

The problem of vehicle re-identification is considered a two-class problem. The actual category of the requested image is considered as a positive category, and the false category is considered as a negative category. The identification results of the network are also divided into positive and negative categories.

*precision* is calculated as shown in Formula (24).


(24) $$\mathrm{precision}=\frac{TP}{TP+FP}$$

The *precision* represents the proportion of samples identified as positive classes in which the actual type is positive.

*AP* (Average Precision) represents average precision.


(25) $$AP=\frac{1}{n}\overset{n}{\underset{i=1}{\sum }}p_{i}$$

In Formula (25), *n* denotes the number of right images returned, $p_{i}$ denotes the corresponding precision of the first correct image.

When there are multiple re-identification objects, we average multiple *AP* values to mAP ;


(26) $$\mathrm{mAP}=\frac{1}{N}\overset{N}{\underset{i=1}{\sum }}{\left(AP\right)}_{i}$$

In Formula (26), *N* denotes the number of re-identified objects. ${\left(AP\right)}_{i}$ means the AP value of the *i* re-identification object.

### 4.4 Ablation experiment

In this section, we investigate the effectiveness of critical components in the mixed sample contrastive learning framework by conducting ablation studies on two different datasets. We introduced HSV features, type features, and the DC module into the network separately. Our proposed methodology aimed to enhance the differentiation between vehicles based on color and type, prompting the model to distinguish vehicles. Additionally, the DC module was utilized to shorten feature distances within image classes and expand feature distances between image classes. The experimental results demonstrated the significant effectiveness of multi-attribute features and the DC module in the context of vehicle re-identification tasks. The accuracy of the multi-attribute features composed of HSV color features and type features, along with the DC module, is presented in Table 2.

**Table 2:** Experimental results of the vehicle classification method.

Algorithm	VeRi776-*mAP*	VeRi-Wild (3000)-*mAP*	VeRi-Wild (5000)-*mAP*
Backbone	42.54	50.16	49.72
Backbone + HSV	52.43	63.51	59.47
Backbone + type	44.67	55.47	53.92
Backbone + HSV + DC	60.61	67.34	63.97
Backbone + type + DC	58.49	62.73	61.35
Final	68.83	71.39	68.42

First, we incorporated the extraction of HSV color features for vehicle recognition into the network. Color is considered as a pivotal attribute for vehicles, enhancing the effectiveness of vehicle re-identification tasks. HSV color features serve to diminish the influence of image brightness on vehicle color recognition while also filtering out high saturation image elements such as windows and backgrounds that could otherwise interfere with color feature identification. Leveraging these color attributes, our model achieved an accuracy of 52.43% on VeRi-776 and 63.51% and 59.47% on VeRi-Wild (3000) and VeRi-Wild (5000), respectively.

Subsequently, vehicle-type features were introduced into the network. Type features assist in distinguishing visually similar vehicles. By leveraging type attributes, our model achieved an accuracy of 44.67% on VeRi-776 and 55.47 and 53.92% on VeRi-Wild (3000) and VeRi-Wild (5000), respectively.

Finally, we incorporated the distance control (DC) module into the network to assess its impact on accuracy. In networks featuring both HSV color and type features, the inclusion of the DC module resulted in our model achieving accuracies of 60.61% and 58.49% for VeRi-776, 67.34% and 63.97% for VeRi-Wild (3000), and 62.73% and 61.35% for VeRi-Wild (5000). The results in the seventh row show that the combined application of HSV color features, type features, and the DC module yields the highest mAP .

### 4.5 Experimental results and analysis

As shown in Table 3, the accuracy of CNN, VGG16, ResNet50, dense network, HSV + CNN, HSV + VGG16, HSV + ResNet50, and HSV + dense network is 83.17%, 86.47%, 91.78%, 90.63%, 88.76%, 92.84%, 95.06%, and 94.24%, respectively. The classification efficiency is 50 n/s , 45 n/s , 63 n/s , 102 n/s , 47 n/s , 40 n/s , 55 n/s , and 94 n/s , respectively. In comparison, the accuracy of our algorithm ranks second, but compared with the first algorithm, the difference is only 0.82%, and the classification efficiency of this algorithm is higher than 39 n/s , so our algorithm is better than HSV+ ResNet50. As for the classification rate, our algorithm, although second, is only 8 n/s lower than the first one (dense connection network), yet the accuracy is 3.61% higher. The optimal accuracy and classification rate in comparative experiments, along with the results of the proposed method in this paper, have been bolded in Table 3. Therefore, in overall consideration, the accuracy and classification efficiency of the proposed algorithm are relatively optimal.

**Table 3:** Experimental results of the vehicle classification method.

Algorithm	Accuracy (%)	Classification efficiency(n/s)
CNN	83.17	50
VGG16	86.47	45
ResNet50	91.78	63
Densely connected network	90.63	**102**
HSV + CNN	88.76	47
HSV + VGG16	92.84	40
HSV + ResNet50	**95.06**	55
HSV + Densely connected network	**94.24**	**94**

Table 4 shows the comparison between the proposed algorithm model and the mainstream re-identification network on the VeRi776 dataset. Table 5 shows the comparison between the proposed algorithm and the mainstream algorithm on the VeRi-Wild dataset, using measures rank−n and mAP .

**Table 4:** Experimental comparison of the VeRi776 dataset.

Models	*mAP* (%)	*rank−1* (%)	*rank−5* (%)
LOMO (Liao et al., 2015)	9.64	25.33	46.48
DGD (Xiao et al., 2016)	17.92	50.70	67.52
GoogLeNet (Yang et al., 2015)	17.81	52.12	66.79
FACT (Liu et al., 2016b)	18.73	51.85	67.16
Siamese Visual (Shen et al., 2017)	29.48	41.12	60.31
PAMAL (Tumrani et al., 2020)	45.06	-	-
MSCL (Yuefeng et al., 2022)	45.90	81.20	-
OIFE (Wang et al., 2017)	48.00	65.92	87.66
VAMI (Zhu et al., 2017)	50.13	77.03	90.82
QD-DFL (Zhu et al., 2020)	51.83	88.50	94.46
VRSDnet (Zhu et al., 2019)	53.45	83.49	92.55
FDA-Net (Lou et al., 2019a)	53.46	84.27	92.43
MV-GAN (Zhang et al., 2021)	61.16	91.06	95.77
VAAG (Tumrani et al., 2023)	63.01	92.20	96.64
VPEN (Meng et al., 2020)	67.98	90.36	94.84
VehicleNet (Zheng et al., 2021)	67.48	90.58	95.47
UFC (Wang et al., 2021)	68.24	91.84	96.73
CTCAL (Yu et al., 2021)	68.65	90.46	95.97
Ours	68.83	92.94	96.88

**Table 5:** Experimental comparison of the VeRi-Wild dataset.

Algorithm	VeRi-Wild (3000)			VeRi-Wild (5000)		
	*mAP* (%)	*rank−1* (%)	*rank−5* (%)	*mAP* (%)	*rank−1* (%)	*rank−5* (%)
GoogLeNet (Liao et al., 2015)	24.27	57.16	75.13	24.15	53.16	71.11
HDC (Yuan et al., 2017)	29.14	57.13	78.93	24.76	49.64	72.28
Unlabled GAN (Zhu et al., 2017)	29.86	58.06	79.60	24.71	51.58	74.42
FDA-Net (Lou et al., 2019a)	35.11	64.03	82.81	29.80	57.82	78.34
FDA-Net (Resnet50)	61.57	73.62	91.23	52.69	64.29	85.39
CTCAL (Yu et al., 2021)	70.35	83.64	92.63	65.73	80.31	90.75
Ours	71.39	86.37	94.39	68.42	83.15	92.93

**Figure 9:**
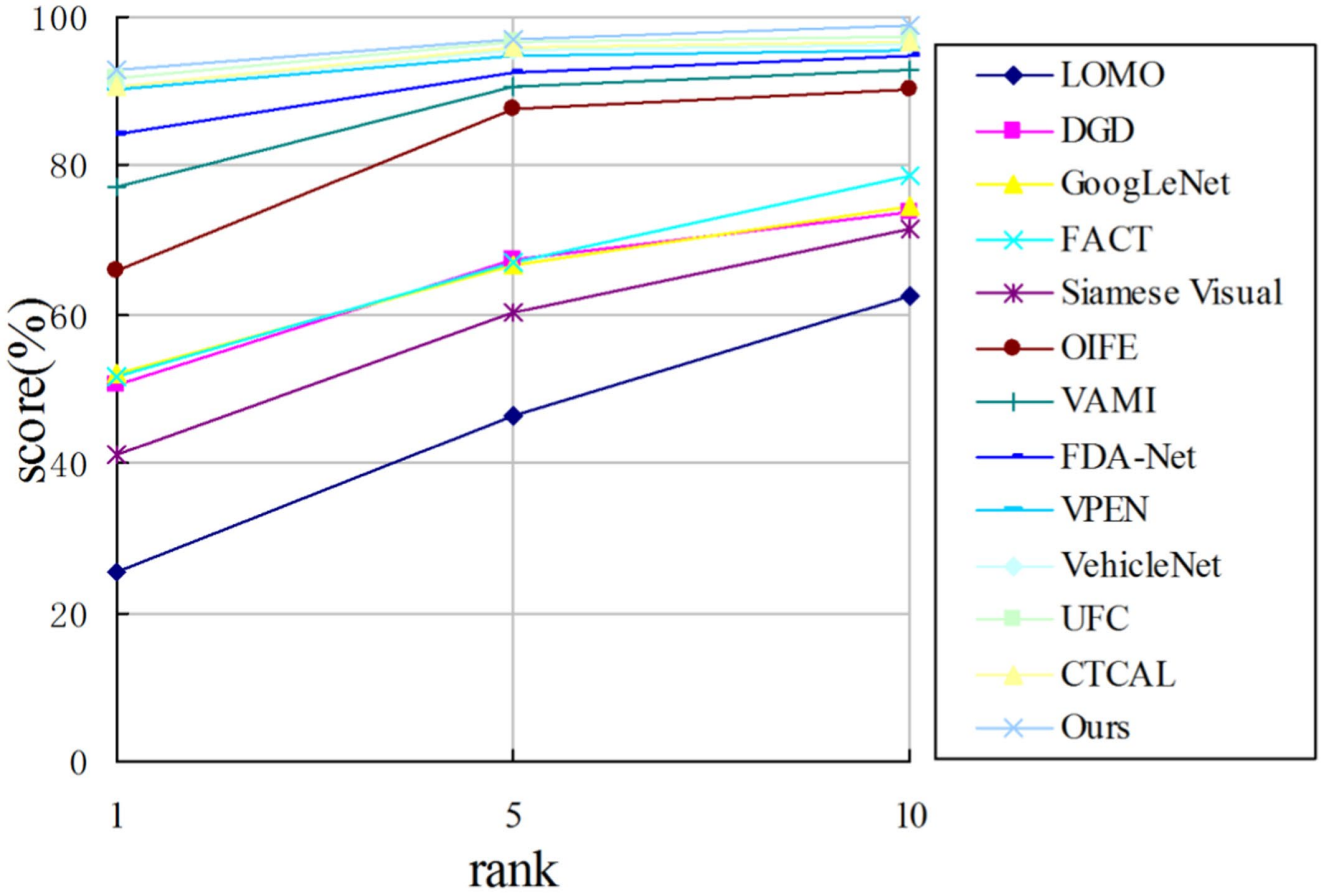
CMC curve on the VeRi776 dataset.

From Table 4, it can be observed that, mAP , rank−1 , and rank−n of this algorithm achieve 68.83, 92.94, and 96.88%, respectively, and each index is the best. As for the second index, the mAP index is higher than the second by 0.18%, rank−1 is higher by 2.84%, rank−5 is higher by 0.15%. As we can observe, this algorithm has the best performance among the above algorithms using VeRi776 dataset as the benchmark. As shown in Figure 9, the probability of classifying the first image output as the correct image is the highest compared with the other images. The advantage of this algorithm is that the hit rate of the model used in this study is relatively high compared with other algorithms.

Table 5 shows the experimental results of six different algorithms on the VeRi-Wild (3000) dataset and the VeRi-Wild (5000) dataset. It is obvious that due to the complex background and the angle of the vehicle images in the VeRi-Wild dataset, the overall performance of the index is lower than that of the VeRi776 dataset.

On the VeRi-Wild (3000) dataset, compared with the second-best CTCAL, the proposed algorithm outperforms by 1.04% in mAP , 2.73% in rank−1 , and 1.76% in rank−5 . In comparison to VAAG, which also utilizes multiple vehicle attributes for vehicle re-identification, the proposed algorithm demonstrates superiority by 2.14% in mAP , 3.10% in rank−1 , and 0.92% in rank−5 .

On the VeRi-Wild (5000) dataset, the proposed algorithm outperforms the second-best CTCAL by 2.69% in the *m*AP metric, 2.84% in the rank−1 metric, and 2.18% in the rank−5 metric.

**Figure 10:**
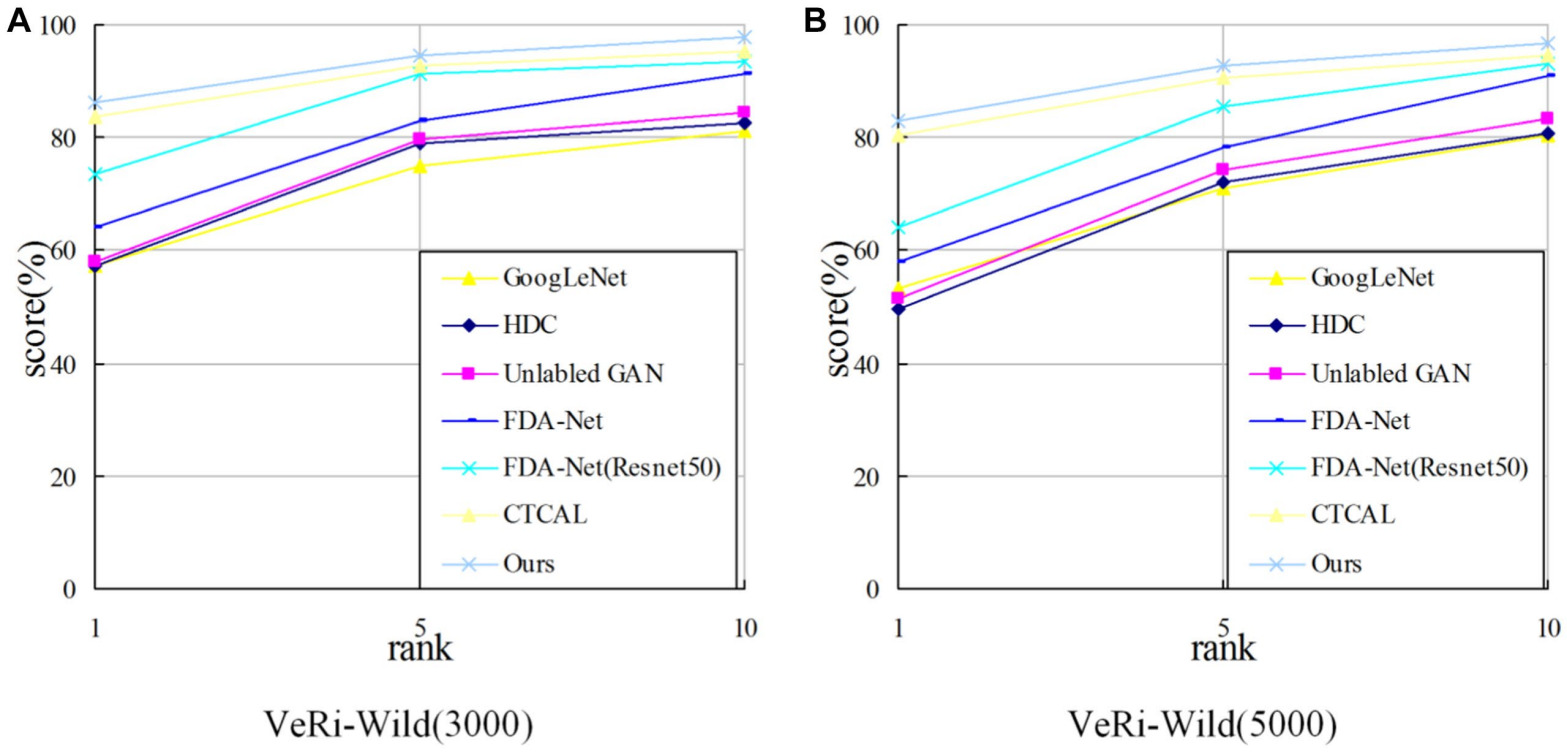
CMC comparison on the VeRi-Wild dataset. **(A)** CMC comparison on the VeRi-Wild (3000); **(B)** CMC comparison on the VeRi-Wild (5000).

As shown in Figure 10, the algorithm in this study outperforms other algorithms in the metric rank−1 . With the increase of *n* value in the metric rank−n , the metric decreases gradually. Together with the experimental data in Table 4, it is proved that the algorithm in this study can distinguish the images with high similarity in the output images (the images in the foreground of the results) and improve the similarity between the classes.

**Figure 11:**
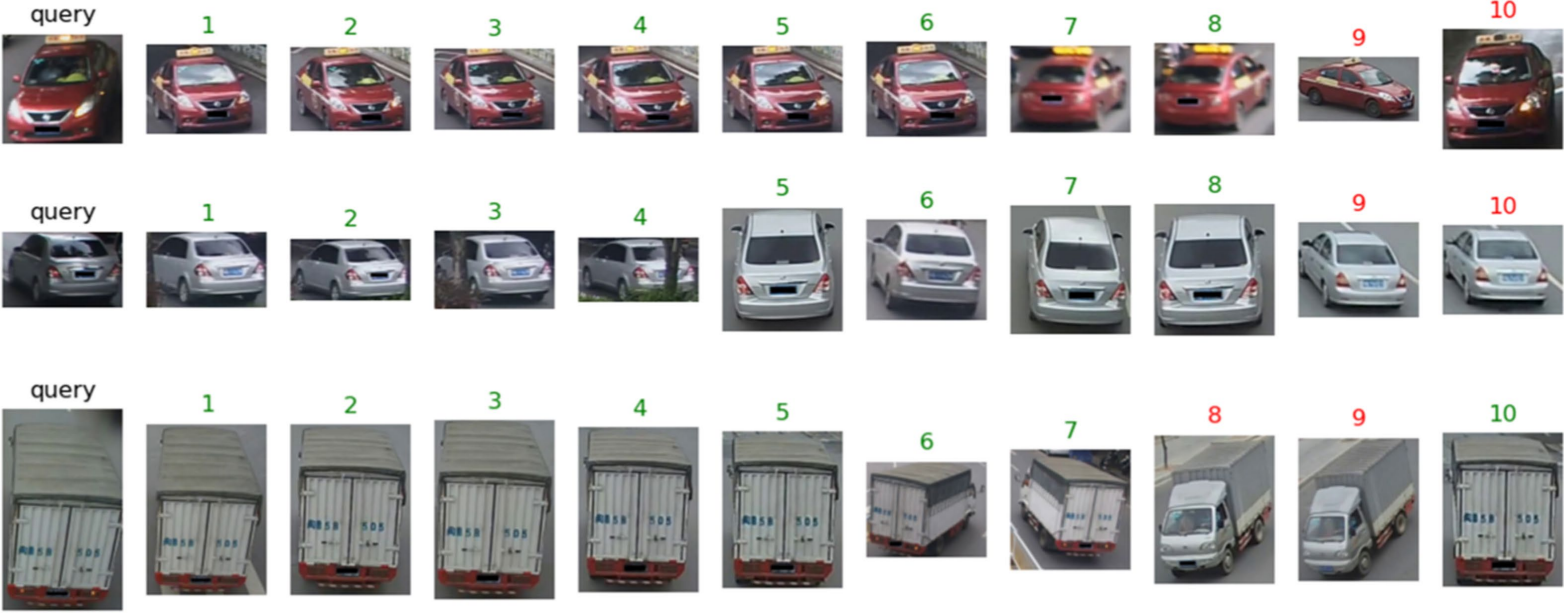
The proposed model results on the VeRi776 dataset. The green numbers and red numbers illustrate the correct and wrong matches.

**Figure 12:**
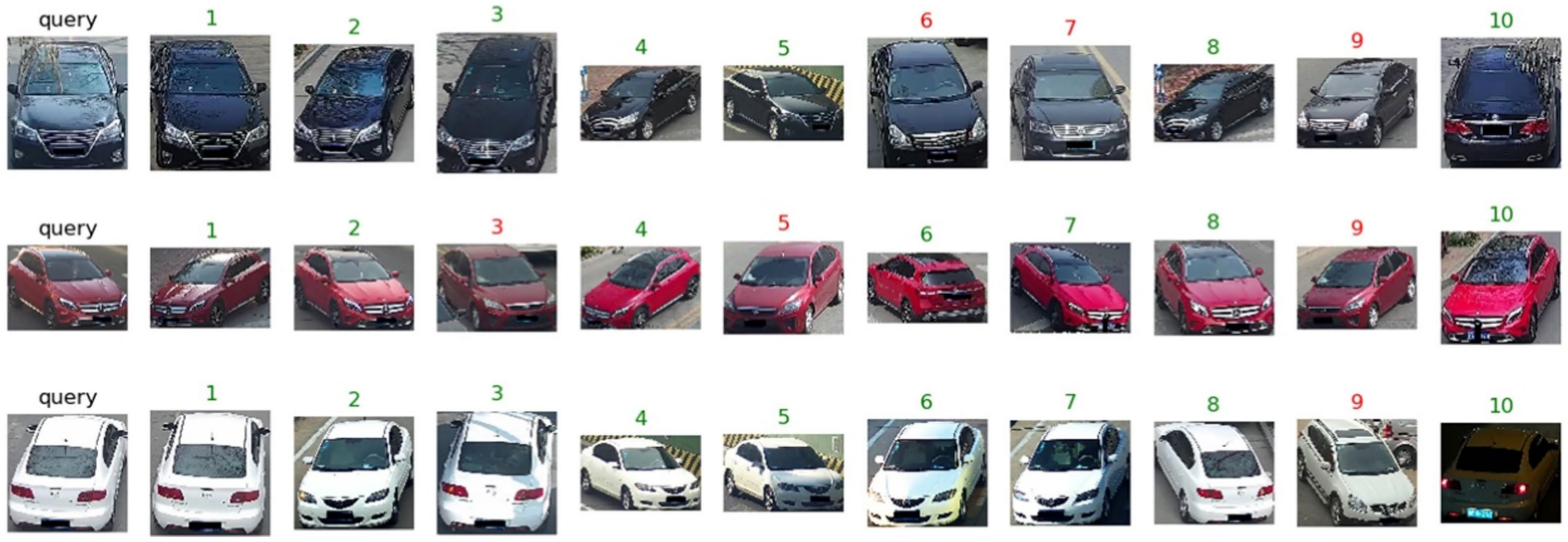
The proposed model results on the VeRi-Wild dataset. The green numbers and red numbers illustrate the correct and wrong matches.

The query images and ranking lists obtained by the final model on the VeRi776 dataset and VeRi-Wild are visually presented in Figures 11, 12. It can be observed that vehicles exhibit different appearances when subjected to varying perspectives, lighting conditions, and occlusions. Even during nighttime driving with illumination and reflection interference, the model can still recognize target images (Figure 12, last row). Overall, the experimental results indicate that the proposed method outperforms existing state-of-the-art multi-attribute-based vehicle re-identification methods.

## 5 Conclusion

In this study, we propose a vehicle re-identification method that integrates a multi-attribute dense connection network with a distance control (DC) module. This model introduces a multi-attribute dense connection mechanism based on HSV color attributes and category attributes in the feature extraction segment of the network, reducing computational complexity. Feature extraction is achieved through multi-dimensional feature-weighted fusion, enhancing both feature extraction and classification accuracy. Furthermore, a method controlling inter-category distances is introduced, employing a DC module as an image distance control module. This module comprises both similar and different DC modules. Based on the target image, features of input images are processed through both similarity and difference DC modules, and the resulting features are then merged into the subsequent network for similarity determination. This module effectively reduces feature distances within image categories while increasing distances between image categories, thereby elevating the accuracy of vehicle re-identification. Experiments for vehicle re-identification are conducted using the VeRi776 dataset, yielding precision and recall values of 68.83 and 92.94%, respectively, surpassing values obtained by other comparative algorithms. Further experiments using the VeRi-Wild (3000) and VeRi-Wild (5000) datasets for vehicle re-identification demonstrate precision values of 71.39% and 68.42% and recall values of 86.37% and 83.15%, respectively, outperforming other algorithms. Experimental results affirm the efficacy of the proposed method in enhancing the accuracy of vehicle re-identification.

## Data availability statement

The data analyzed in this study is subject to the following licenses/restrictions: the production personnel of this dataset ask for your information only to make sure the dataset is used for non-commercial purposes. They will not give it to any third party or publish it publicly anywhere. Requests to access these datasets should be directed to VeRi776 datasets, https://vehiclereid.github.io/VeRi/ and VeRi-Wild datasets, https://github.com/PKU-IMRE/VERI-Wild.

## Author contributions

XS: Methodology, Software, Writing – review & editing. YC: Methodology, Software, Writing – original draft. YD: Methodology, Software, Writing – original draft. YW: Methodology, Software, Writing – original draft. JZ: Methodology, Software, Writing – original draft. BS: Supervision, Writing – review & editing. LL: Supervision, Writing – review & editing.
